# Supplementary material for: Contribution of Mitochondrial DNA Heteroplasmy to the Congenital Cardiac and Palatal Phenotypic Variability in Maternally Transmitted 22q11.2 Deletion Syndrome
Source: Genes (Basel). 2021 Jan 13;12(1):92. doi: 10.3390/genes12010092 (PMC7828421; doi:10.3390/genes12010092)
Supplement: Supplementary file 1 [file genes-12-00092-s001.pdf]

genes

Supplementary Information

Figure S1. Depth distribution per individual. Samples are colored according to familial relationship.

Figure S2. Depth distribution per mtDNA position. Each position in the human mtDNA (16,569 positions) is represented by a boxplot according to the colors indicated in the legend.

Figure S3. Neighbor joining tree reflecting maternal relationships. The tree has been rooted with the Reconstructed Sapiens Reference Sequence (RSRS).

Figure S4. Minor allele frequency distribution of samples with heteroplasmic sites. MAF: Minor allele frequency.

Figure S5. Contamination assessment for samples DG33 and DG224. Samples DG33 and DG224, both had 5 heteroplasmic sites each with apparently non-random minor allele frequencies (the five sites had very similar AF), but they do not cluster with unrelated sequences.

Table S1. Allele frequency change (ΔAF).

Table S2. mtDNA haplogroups. We confirmed maternal relationships between mother-child pairs based on their haplogroup. Conf: posterior probability of haplogroup assignment.

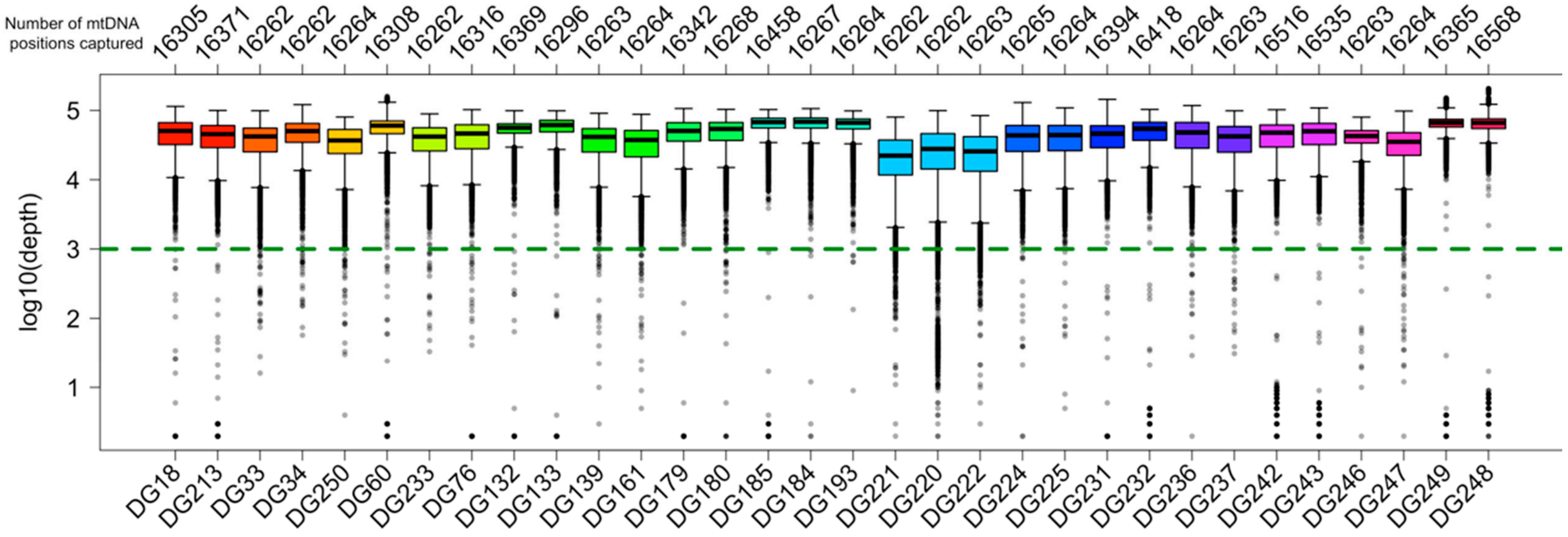

Figure S1. Depth distribution per individual. Samples are colored according to familial relationship.

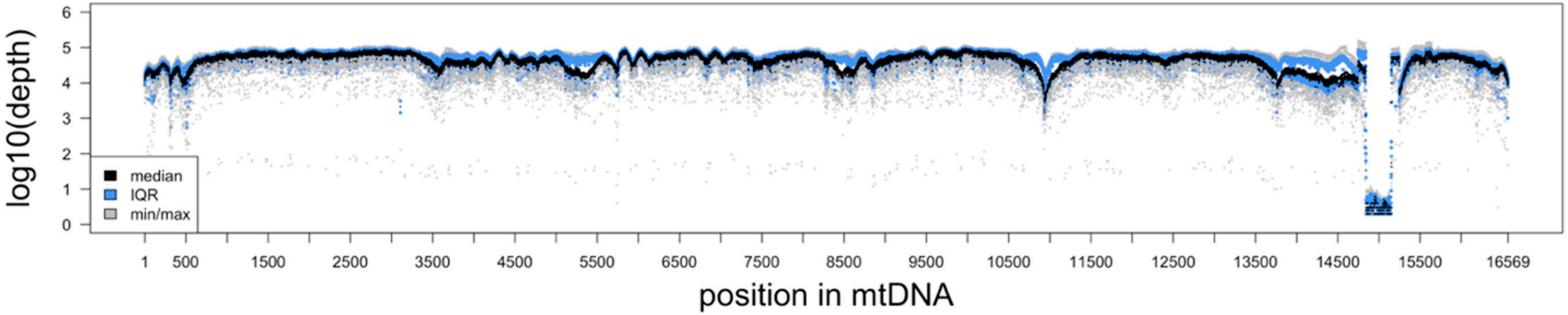

Figure S2. Depth distribution per mtDNA position. Each position in the human mtDNA (16,569 positions) is represented by a boxplot according to the colors indicated in the legend.

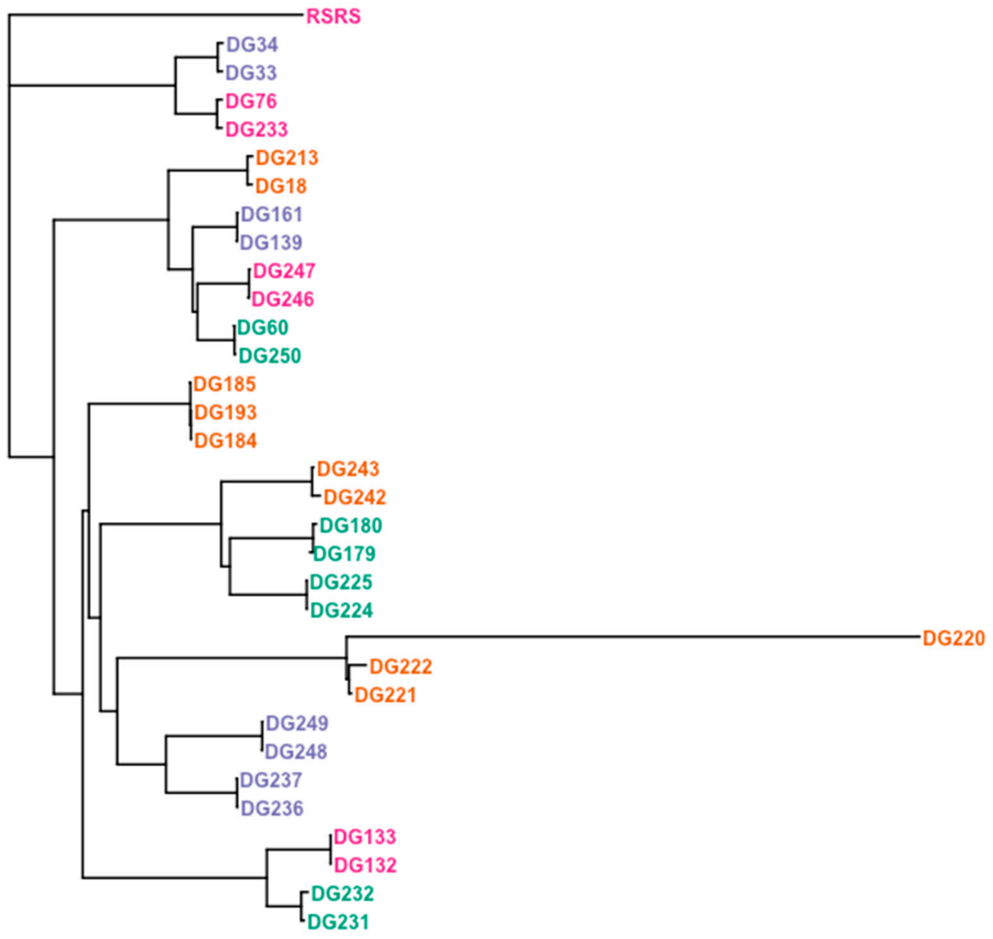

Figure S3. Neighbor joining tree reflecting maternal relationships. Pairwise distance. The tree has been rooted with the Reconstructed Sapiens Reference Sequence (RSRS, Behar et al, 2012).

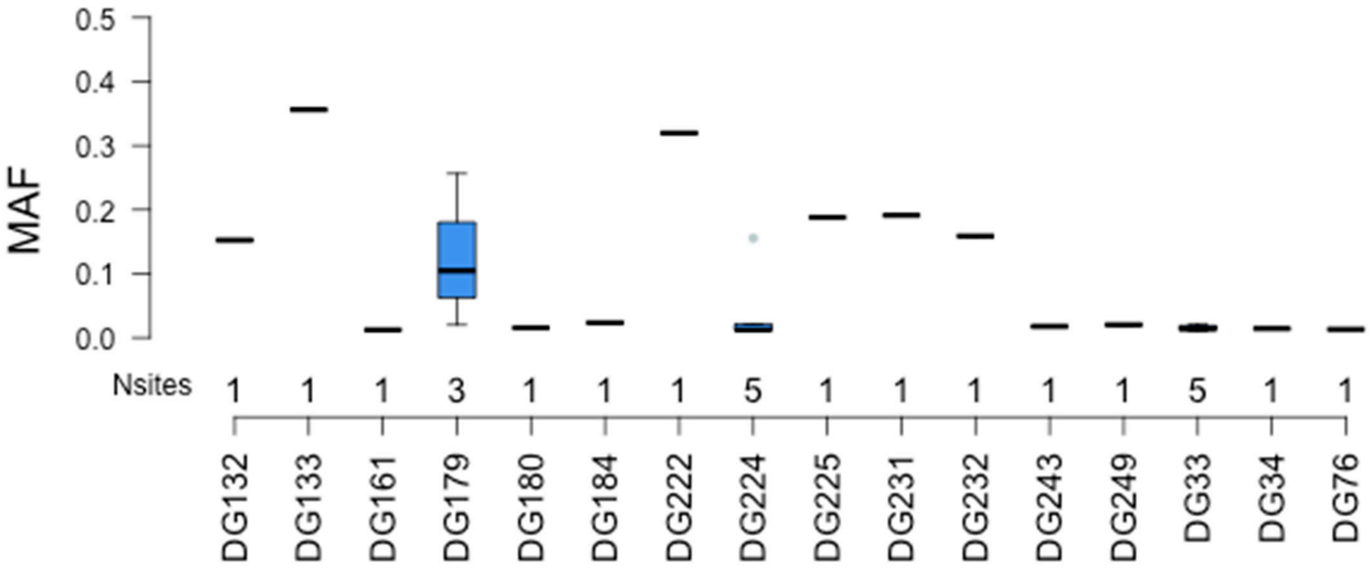

Figure 4. Minor allele frequency distribution of samples with heteroplasmic sites. MAF: Minor allele frequency.

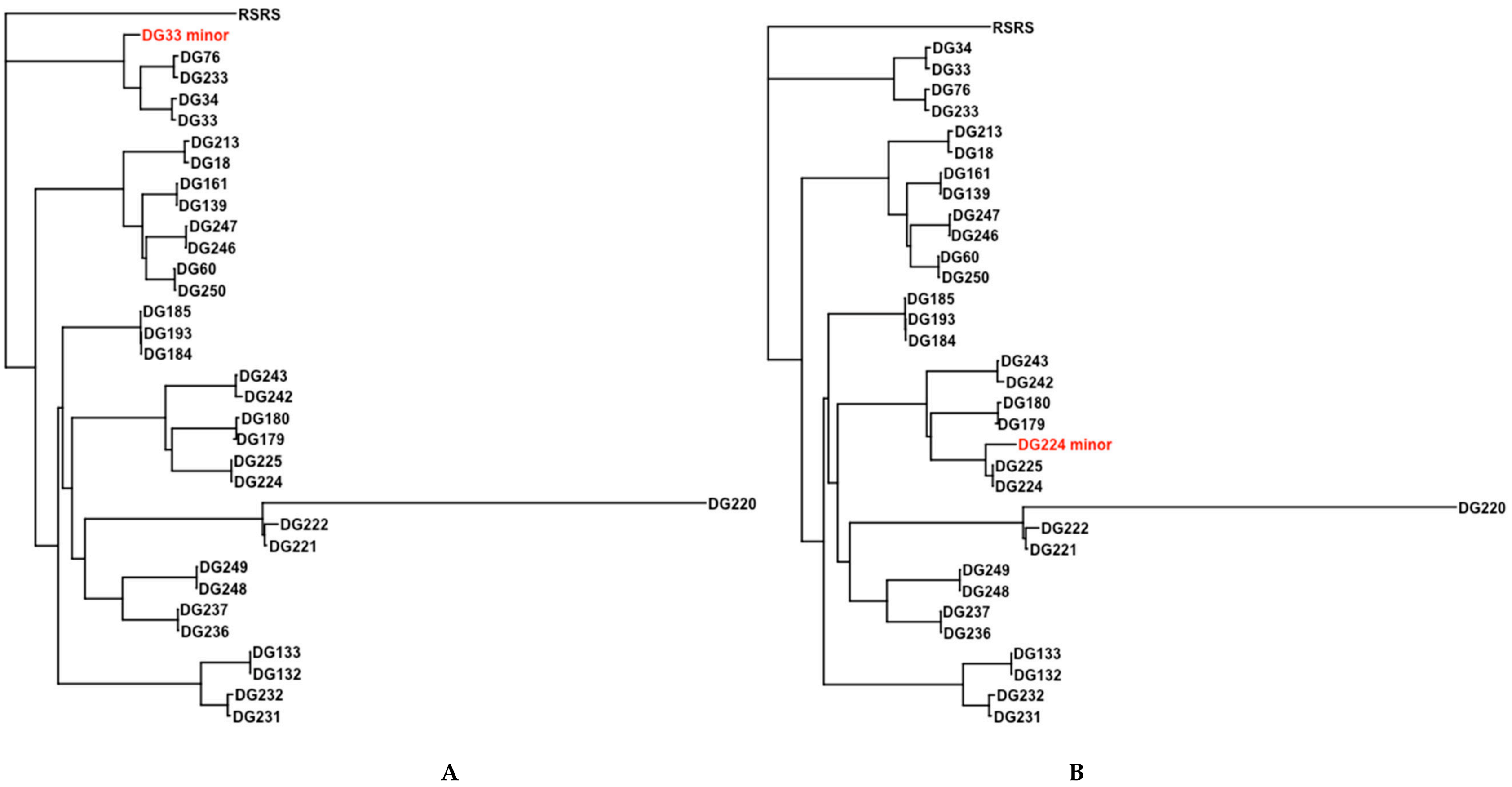

Figure S5. Contamination assessment for samples DG33 and DG224. Samples DG33 (**A**) and DG224 (**B**), both had 5 heteroplasmic sites each with apparently non-random minor allele frequencies (the five sites had very similar AF), but they do not cluster with unrelated sequences.

Table S1. Allele frequency change (ΔAF) between a mother and her child for transmissions. mid: mother id. cid: child id. pos: position. nt: allele. mc: mother allele count. md: mother site depth. AFm: Allele frequency mother. cc: child allele count. cd: child site depth. AFc: Allele frequency child. ΔAF: Delta allele frequency (child-mother). ht: heart phenotype. pl: palate phenotype. * de novo mutation not considered for the analyses.

Pair	mid	cid	pos	nt	mc	md	Afm	cc	cd	Afc	ΔAF	ht	pl	
2	DG34	DG33	6802	G	529	37,167	1.42×10^{-2}	1	43,112	2.30×10^{-5}	-1.42×10^{-2}	con	con	
	DG34	DG33	10,873	T	7	9519	7.35×10^{-4}	170	11,859	1.43×10^{-2}	1.36×10^{-2}	con	con	*
	DG34	DG33	14,668	C	15	34,435	4.36×10^{-4}	54	4713	1.15×10^{-2}	1.10×10^{-2}	con	con	
	DG34	DG33	14,783	T	11	23,194	4.74×10^{-4}	143	13,425	1.07×10^{-2}	1.02×10^{-2}	con	con	
	DG34	DG33	15,301	G	1	50,276	2.00×10^{-5}	194	10,341	1.88×10^{-2}	1.87×10^{-2}	con	con	*
	DG34	DG33	15,326	G	2	47,401	4.20×10^{-5}	307	14,117	2.17×10^{-2}	2.17×10^{-2}	con	con	*
4	DG76	DG233	15,786	C	867	66,261	1.31×10^{-2}	0	62,238	0.00	-1.31×10^{-2}	dis	dis	
5	DG133	DG132	9507	C	21847	61,348	3.56×10^{-1}	8407	55,188	1.52×10^{-1}	-2.04×10^{-1}	dis	dis	
6	DG161	DG139	5492	C	362	29,462	1.23×10^{-2}	5	30,038	1.66×10^{-4}	-1.21×10^{-2}	dis	dis	
7	DG180	DG179	150	T	298	19,275	1.55×10^{-2}	4900	19,122	2.56×10^{-1}	2.41×10^{-1}	dis	con	
	DG180	DG179	1316	C	2	24,450	8.20×10^{-5}	2255	21,577	1.05×10^{-1}	1.04×10^{-1}	dis	con	*
	DG180	DG179	5054	A	37	68,532	5.40×10^{-4}	1410	70,310	2.01×10^{-2}	1.95×10^{-2}	dis	con	
8	DG184	DG185	15,591	A	630	26,908	2.34×10^{-2}	20	39,996	5.00×10^{-4}	-2.29×10^{-2}	dis	dis	
9	DG184	DG193	15,591	A	630	26,908	2.34×10^{-2}	19	32,685	5.81×10^{-4}	-2.28×10^{-2}	dis	dis	
10	DG220	DG221	16,290	C	6712	7398	9.07×10^{-1}	6713	6716	1.00	9.23×10^{-2}	con	dis	
11	DG220	DG222	16,290	C	6712	7398	9.07×10^{-1}	4390	13,735	3.20×10^{-1}	-5.88×10^{-1}	con	dis	
12	DG225	DG224	6190	A	117	17,184	6.81×10^{-3}	94	8443	1.11×10^{-2}	4.33×10^{-3}	con	con	
	DG225	DG224	10,075	C	0	80,165	0.00	1636	77,194	2.12×10^{-2}	2.12×10^{-2}	con	con	*
	DG225	DG224	12,315	A	10	46,357	2.16×10^{-4}	509	44,701	1.14×10^{-2}	1.12×10^{-2}	con	con	
	DG225	DG224	12,457	A	25	7323	3.41×10^{-3}	34	2796	1.22×10^{-2}	8.75×10^{-3}	con	con	
	DG225	DG224	16,182	A	432	2299	1.88×10^{-1}	423	2721	1.55×10^{-1}	-3.25×10^{-2}	con	con	
13	DG232	DG231	4136	G	6158	38,779	1.59×10^{-1}	6499	34,006	1.91×10^{-1}	3.23×10^{-2}	dis	con	
15	DG243	DG242	15,431	A	689	38,994	1.77×10^{-2}	15	36,189	4.14×10^{-4}	-1.73×10^{-2}	dis	con	
17	DG248	DG249	9941	G	6	85,309	7.00×10^{-5}	1615	79,897	2.02×10^{-2}	2.01×10^{-2}	dis	dis	*

Table S2. mtDNA haplogroups. We confirmed maternal relationships between mother-child pairs based on their haplogroup. Conf: posterior probability of haplogroup assignment.

Pair	Mother	Child	Hap_mother	Conf_mother	Hap_child	Conf_child
1	DG213	DG18	A2 + (64) + @153	0.87	A2 + (64)+ @153	0.88
2	DG34	DG33	D1j1a	0.94	D1j1a	0.93
3	DG60	DG250	A2 + (64)	0.97	A2 + (64)	0.97
4	DG76	DG233	D1j1a1	0.98	D1j1a1	0.97
5	DG133	DG132	T2b3 + 151	0.93	T2b3 + 151	0.93
6	DG161	DG139	A2 + (64)	0.93	A2 + (64)	0.93
7	DG180	DG179	B2	0.80	B2	0.80
8	DG184	DG185	H3ao1	0.93	H3ao1	0.93
9	DG184	DG193	H3ao1	0.93	H3ao1	0.93
10	DG220	DG221	U1a1a3	0.69	U1a1a3	0.90
11	DG220	DG222	U1a1a3	0.69	U1a1a3	0.89
12	DG225	DG224	B2	0.78	B2	0.78
13	DG232	DG231	T2b	0.95	T2b	0.96
14	DG237	DG236	U5b1 + 16189 + @16192	0.96	U5b1 + 16189 + @16192	0.96
15	DG243	DG242	B2b3a	0.91	B2b3a	0.91
16	DG247	DG246	A2 + (64)	0.93	A2 + (64)	0.93
17	DG248	DG249	U5a1a1	0.96	U5a1a1	0.96
